# Regulation of expression of O^6^-methylguanine-DNA methyltransferase and the treatment of glioblastoma (Review)

**DOI:** 10.3892/ijo.2015.3026

**Published:** 2015-05-29

**Authors:** GIULIO CABRINI, ENRICA FABBRI, CRISTIANA LO NIGRO, MARIA CRISTINA DECHECCHI, ROBERTO GAMBARI

**Affiliations:** 1 Department of Pathology and Diagnostics, University Hospital, Verona, Italy; 2 Department of Life Sciences and Biotechnology, University of Ferrara, Ferrara, Italy; 3 Department of Oncology, S. Croce and Carle Teaching Hospital, Cuneo, Italy

**Keywords:** MGMT, glioblastoma, glioma, temozolomide, microRNA, peptide nucleic acids, locked nucleic acids, methylation, pyrosequencing

## Abstract

O-6-methylguanine-DNA methyltransferase (MGMT) is an abundantly expressed nuclear protein dealkylating O^6^-methylguanine (O^6^-MG) DNA residue, thus correcting the mismatches of O^6^-MG with a thymine residue during DNA replication. The dealkylating effect of MGMT is relevant not only in repairing DNA mismatches produced by environmental alkylating agents promoting tumor pathogenesis, but also when alkylating molecules are applied in the chemotherapy of different cancers, including glioma, the most common primary tumor of the central nervous system. Elevated MGMT gene expression is known to confer resistance to the treatment with the alkylating drug temozolomide in patients affected by gliomas and, on the contrary, methylation of MGMT gene promoter, which causes reduction of MGMT protein expression, is known to predict a favourable response to temozolomide. Thus, detecting expression levels of MGMT gene is crucial to indicate the option of alkylating agents or to select patients directly for a second line targeted therapy. Further study is required to gain insights into MGMT expression regulation, that has attracted growing interest recently in MGMT promoter methylation, histone acetylation and microRNAs expression. The review will focus on the epigenetic regulation of MGMT gene, with translational applications to the identification of biomarkers predicting response to therapy and prognosis.

## 1. Role of MGMT in cancer

O^6^-methylguanine (O^6^-MG) is one of the major mutagenic and carcinogenic lesions in DNA induced by alkylating mutagens, because of its preference for pairing with thymine instead of cytosine during DNA replication. O^6^-methylguanine-DNA methyltransferase (MGMT) is a ubiquitously expressed nuclear enzyme which removes alkyl groups from the O^6^-position of O^6^-MG (1). Each single alkyl group removed from O^6^-MG is transferred to a cysteine residue within the active site of MGMT in a stoichiometric second-order reaction, implying the inactivation of one molecule of MGMT enzyme for each alkyl group removed from methylguanine, a process termed suicide inhibition (2). Consequently, the efficiency of O^6^-MG repair is limited by the number of molecules of MGMT enzyme available, also considering that the dealkylating function of MGMT does not possess redundant or alternative pathways. MGMT-mediated removal of alkyl groups from O^6^-MG is also relevant in alkylating chemotherapy of glioma, such as with temozolomide and nitrosourea derivatives. Massive DNA alkylation produced by temozolomide causes base mispairing (3,4). If O^6^-MG is not repaired because of low MGMT expression, O^6^-MG forms a base pair with thymine. The mismatched O^6^-MG to thymine base pair is recognized by the pathway involving the repair proteins MLH1, MSH2, MSH6 and PMS2, resulting in futile cycles of repair that lead to cell cycle arrest and cell death (5). On the contrary, the methylation damage produced by temozolomide can be reversed by MGMT, as its DNA repairing activity provides resistance against the cytotoxic effects of guanine methylation. As already shown in clinical trials in patients affected by glioma that have been treated with the alkylating drug temozolomide, whose response to therapy is significantly ameliorated when MGMT expression is reduced because of promoter methylation (6).

## 2. Nuclear transcription factors regulating the expression of MGMT gene

MGMT gene (ID: 4255, NCBI Ref Seq NM_002412.3) has been cloned (1) and mapped to chromosome 10 in the cytogenetic location 10q26.3, where it spans 15 Kb in length (7). So far 142 single nucleotide variants of MGMT gene have been reported. Transcript of 1265 nucleotides is organized in 5 exons encoding a protein of 238 amino acid residues. A CpG island of 762 base pairs that includes 97 CpG dinucleotides spans the proximal promoter region and the first exon. Different transcription factors have been found to activate the transcription of MGMT gene, including Sp1 (8), NF-κB (9), CEBP (10) and AP-1 (10,11). The cellular tumor antigen p53 has been associated with repression of MGMT transcription (12), possibly via sequestering the Sp1 nuclear transcription factor (13).

**Figure 1:**
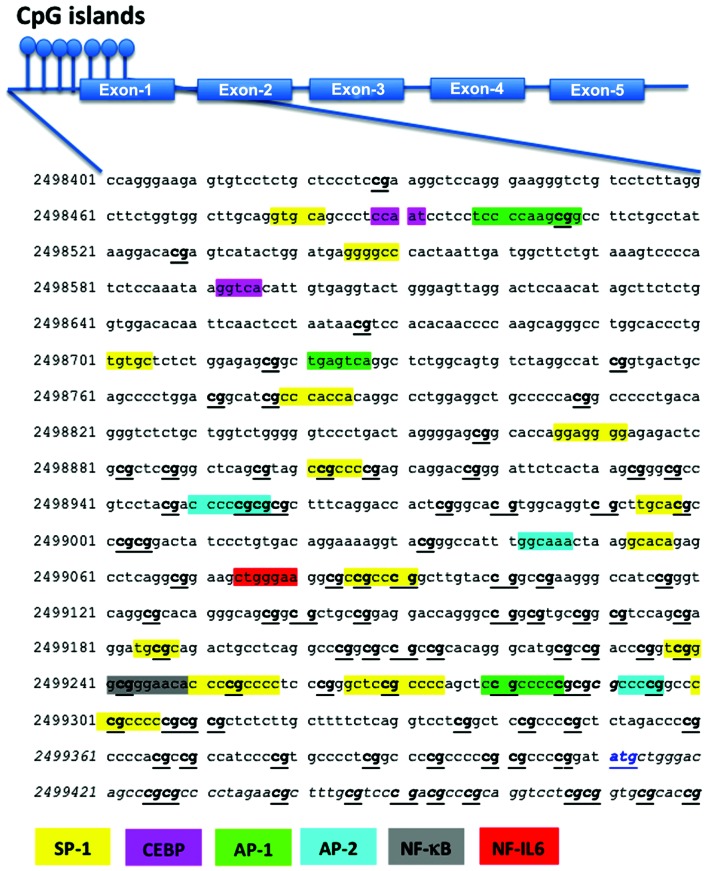
MGMT promoter: schematic of transcription factors and methylation islands. Schematic representation of the CpG dinucleotides (bold underlined) and the putative consensus sequences of the major nuclear transcription factors involved in MGMT transcriptional activation as identified *in silico* by Transcription Elements Search System (TESS) analysis (http://www.cbil.upenn.edu/tess). Color boxes indicate the position of the nuclear transcription factors SP-1, CEBP, AP-1, AP-2, NF-κB and NF-IL6.

*In silico* analysis on the putative consensus sequences for the binding of nuclear transcription factors in MGMT promoter sequence reveals further nuclear factors potentially involved in activating MGMT transcription, for instance AP-2, NF-IL6, and ER-α, besides Sp1, AP-1 and c/EBP, as shown in Fig. 1. As some of these consensus sequences include CpG dinucleotides, question has arisen on whether methylation of CpG dinucleotides in key consensus sequences could hinder the binding of the corresponding transcription factors, thus reducing the transcriptional activation of the MGMT gene. For instance, this inhibiting mechanism has been evidenced for Sp1-dependent, but not NF-κB-dependent activation of MGMT transcription (8,9), thus keeping the hypothesis open for other transcription factors. Noteworthy, MGMT expression has been found heterogeneous within histological sections of gliomas, being higher in the inner core of the tumor than in the periphery (14).

Further analyses correlated the high expression of MGMT with the presence of a hypovascular central core of the tumor, where activation of the hypoxia inducible factor (HIF)-1α will in turn promote expression of MGMT, particularly in the hypoxic glioma stem cells niches (14,15). The MGMT/HIF-1α regulatory axis has been further confirmed, since the expression of the bone morphogenetic protein 2 (BMP2) has been shown to downregulate MGMT expression through HIF-1α-dependent downregulation (16). Moreover, hypoxia intervenes further on MGMT function as HIF-1α is known to induce also the expression of the hypoxia-inducible and steroid-inducible N-myc downstream regulated gene 1 (NDRG1) protein, which binds to and stabilizes MGMT protein (17). In summary, although Sp1, AP-1, CEBP, NF-κB and HIF-1α as single transcription factors have been proved to activate MGMT gene regulation (8–11), the understanding of the synergy within these transcription factors in activating MGMT expression and the composition of the MGMT promoter enhanceosome need further investigation.

## 3. Effect of histone modifications

Epigenetic modifications of MGMT gene have been found to play a relevant role in MGMT expression in the context of cancer. Histone acetylation and methylation has been extensively investigated in relation to MGMT expression, in different cancer models including gliomas (18–24). Acetylation of lysine residues on histones H3 and H4 (H3Ac and H4Ac), that are associated with open chromatin and active transcription, has been found elevated in cell lines expressing high level of MGMT, suggesting a role for these histone modifications (18). On the contrary, di-methylation of lysine 9 of histone 3 (H3me2K9) has been found relevant in silencing MGMT expression (20).

The relevance of the role of histone acetylation on MGMT expression has been recently confirmed by testing *in vitro* the effect of the histone deacetylase (HDAC) inhibitor suber-oylalanide hydroxamic acid (SAHA), which increased MGMT expression, thus strengthening resistance to the alkylating agent temozolomide (22). However, considering the multiplicity of the target genes that may be modulated by HDAC inhibitors, these drugs could have opposite effects when used in chemotherapy. For instance, the treatment of U251 glioma cells with the HDAC inhibitor LBH589 increased the sensitivity to temozolomide. This could be explained with an increased expression of the heat shock protein 90 (HSP90), which in turn induced downregulation of expression of the epidermal growth factors receptor (EGFR) and the phosphoprotein p-Akt. This could be responsible for an increase of the pro-apoptotic effect of temozolomide, independently of the levels of expression of MGMT (23).

**Figure 2:**
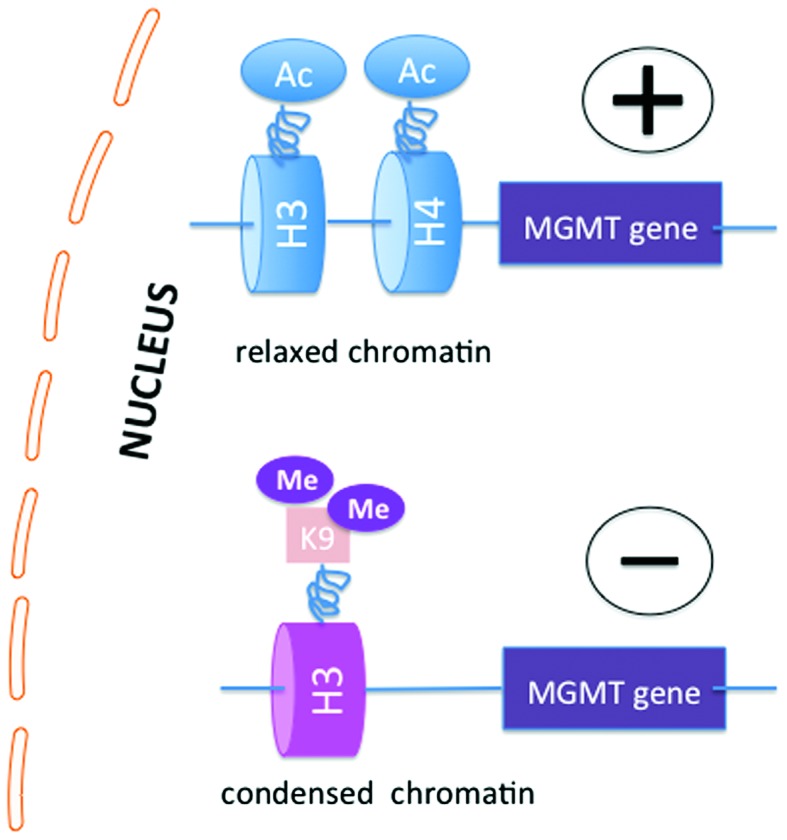
Effect of histone modification on MGMT gene expression. Acetylation of histones H3 and H4 promotes MGMT transcription (18), whereas di-methylation of lysine 9 on histone H3 represses MGMT transcription (20).

The major role of histone in the regulation of expression of MGMT is summarized in Fig. 2. Of note, histone acetylation and the expression levels of HDAC are therefore increasing in relation to the regulation of expression of MGMT, consequently improving the response to therapy and the overall prognosis of patients affected by glioma (24).

## 4. MGMT promoter methylation

Methylation of MGMT promoter is found in 40% of cancer types such as glioma and colorectal cancer and in 25% of non-small cell lung carcinoma, lymphoma and head and neck carcinoma (25). As promoter methylation is one of the major post-transcriptional mechanisms reducing protein expression, the methylation of CpG sites in the promoter and the overall extent of methylation could affect the levels of expression of the protein. Therefore, expression of MGMT protein is significantly reduced in MGMT-methylated cancer cells, as detected by immunohistochemistry (25), and the levels of expression of MGMT protein have been associated with the efficacy of response of cancer cells to alkylating drugs in glioma tumor models in rodents *in vivo* (26,27). All these pieces of evidence prompted investigations on the relation between MGMT promoter methylation and the response to chemotherapy with alkylating drugs in patients affected by gliomas (6). This original observation was soon extended into a large series of clinical investigations (28–84) in order to assess the potency of MGMT methylation as a predictive marker in relation to alkylating therapy in different conditions of adult and pediatric gliomas, of both high and low grade according to WHO classification (85).

The relevance of MGMT methylation as a biomarker has been strengthened by the widely accepted application of the consensus reached in Phase III clinical trials jointly conducted by European and North-American research networks, that were summarized in the so-termed ‘Stupp protocol’ for the first line treatment of patients affected by gliomas, which includes the post-surgery association of radiotherapy and temozolomide (47). The predictive value of MGMT methylation in response to temozolomide has reached over the years an overall confirmatory consensus, such that analysis of MGMT methylation has been included to stratify patients enrolled in major multicenter international clinical trials (38,47,58,60,71,74,75,86) and leading recommendations have been stated on how to treat patients affected by glioma, where the analysis of MGMT methylation is assumed as one of the key decision points in the therapeutic flow-chart (87). Besides the overall confirmatory consensus, different issues have been considered both to interpret the role and to improve the predictive role of MGMT methylation as clinical biomarker. For instance, the overall survival and the progression-free survival of patients treated with temozolomide is related to the overall level of methylation of the MGMT promoter, assessed by quantitative methylation-specific techniques, as highly methylated samples are significantly associated with the best prognosis (50). However, within the 97 CpG dinucleotides identified in the CpG island of the MGMT proxymal promoter, the extent of methylation can be variable, with different effects on the degree of gene silencing (18).

Moreover, methylation of a specific CpG dinucleotide of the CpG island could have different impact on transcriptional downregulation. For instance, binding of the methyl-CpG binding (MCB) protein 2 (MeCP2) to methylated CpG islands have been found relevant in silencing MGMT expression (20). MGMT methylation could interfere with the activatory role of the transcription factor Sp1 (8), but the identification of the specific CpG dinucleotides involved has not been cleared. The expression of MGMT protein is heterogeneous within the glioma tissue. For instance, MGMT promoter methylation is not different among the concentric layers of glioblastoma specimens (15,88) whereas the core of the glioma often presents higher expression of MGMT protein than the peripheral areas (88), possibly because of the transcriptional activation of expression mediated by hypoxia and activation of HIF-1α pathway (14,15). Thus, MGMT promoter methylation is a very relevant, but not a unique mechanism, to regulate the expression of MGMT and the response to therapy with alkylating agents (89).

## 5. MicroRNAs in MGMT expression regulation

MicroRNAs (miRs) (www.mirbase.org) belong to a family of small (19 to 25 nucleotides in length) noncoding RNAs that target specific sequences of mRNAs thereby regulating gene expression (90,91), causing translational repression or mRNA degradation, depending on the degree of complementarities between miRs and the target sequences (92,93). Although *in silico* analysis of the 3′-UTR of the MGMT gene has revealed several potential sequences that could be a site for interaction of miRs (94), miR-dependent regulation of MGMT expression is presently under intensive investigation. Different studies have been recently conducted in order to associate modulation of expression of specific miRs with the response to temozolomide in patients affected by glioblastoma and in experimental cell models *in vitro* (95–127). However, only some of these investigations can be directly related to the therapeutic axis between temozolomide and MGMT expression.

A genome-wide analysis of expression of 1,146 miRs performed in tissue samples obtained from 82 glioblastoma specimens was correlated with overall survival and further validated in The Cancer Genome Atlas (TCGA) dataset, which includes 424 glioma samples (100). Comparative analysis evidenced the miR-181d expression in glioma tissues as inversely correlated with a favourable prognosis in these patients and that the favourable effect of miR-181d is, at least partially, related to its effect in downmodulating MGMT mRNA expression (100). Furthermore, miR-181d expression inversely correlated with that of MGMT mRNA in glioma tissue specimens; moreover, overexpression of miR-181d in A1207, LN340 and T98G glioblastoma cell lines reduced MGMT mRNA levels and conferred pro-apoptotic sensitivity to temozolomide (100).

Additional information on miR-dependent regulation of MGMT expression has been provided by an investigation starting from a bioinformatics analysis in the TCGA database related to glioblastoma (128), aimed to search inverse correlation between miRs levels and MGMT mRNA, taking into account also the contribution of the MGMT promoter methylation (106). The bioinformatics analysis confirmed the role of miR-181d and found miR-767-3p and miR-648 as novel potential regulators of MGMT gene expression (106). Validation in glioma tissues *ex vivo* and in experimental glioblastoma cell models *in vitro* supported the bioinformatics analyses and indicated that downregulation of MGMT expression by miR-181d and miR-767-3p is due to degradation of the MGMT mRNA whereas miR-648 affects MGMT protein translation (106). Experiments performed *in vitro* confirmed that the overexpression of these three miRs reduces MGMT protein expression and confers sensitivity to temozolomide (106). A third contribution was focused on the paralogues miR-221 and miR-222 (114), known to be highly expressed in glioblastoma tissues (128,129). They have been found to downregulate MGMT expression and to confer increased sensitivity to temozolomide (114). These results need to be confirmed, since a pro-apoptotic effect of glioblastoma cell lines was obtained using antagomiR molecules against miR-221 and the expression of the 221/222 cluster was associated to chemio- and radio-resistance (130–133).

A fourth major contribution was obtained by testing significant reduction of MGMT protein expression with a genome-wide miR screening performed by transfecting 885 known miRs into the T98G glioblastoma cell line, which is characterized by high levels of the MGMT protein (122). The miRs identified by the first screening were validated by *in silico* analysis of putative binding sites in the 3′-UTR region of MGMT gene utilizing different algorithms, which restricted the potentially relevant miRs to a limited series, that was verified for inverse correlation of expression with MGMT in the Chinese Cancer Genome Atlas, then tested in LN340 glioblastoma cell line *in vitro* (122). Some of the previously reported miRs (such as miR-221, -222 and -648) did not consistently downregulate MGMT expression in LN340 cells, whereas miR-181d and miR-767-3p had a positive effect (122).

In addition, the novel regulatory miR-603, which directly interacts with the 3′-UTR region of MGMT gene, produced a 6-fold decrease of both MGMT mRNA and protein upon transfection in glioblastoma cell line *in vitro* and sensitized glioblastoma cells to temozolomide (122). Therefore, although considering that temozolomide sensitivity of glioblastoma can be modulated by different miRs independently of the regulation of MGMT expression, six miRs have been reported to be involved in the regulation of MGMT protein expression either by degrading MGMT mRNA, namely miR-181d, -767-3p, -221, -222, -603 (100,106,114,122), or affecting the MGMT protein translation, such as miR-648 (106). Considering that this series of miRs have been identified with different approaches and that the inverse correlation expected between MGMT expression and the expression of each miR is not usually characterized by very high and significant correlation coefficient, possibilities are open that further miRs will be revealed as regulators of MGMT expression and that the MGMT downregulation might require the synergy of action of these and/or other miRs, which presently requires further investigation.

## 6. MGMT expression as predictive biomarker

Inactivation of expression of MGMT gene as an effect of MGMT promoter methylation has been found as a relevant predictive biomarker of the response to the alkylating drug temozolomide in patients affected by glioma (6) and the original observation was basically confirmed by several replication studies (28–84). These studies raised the question on the most reliable method to evaluate MGMT expression, either directly or indirectly, in relation to its clinical predictivity to alkylating therapy of gliomas. MGMT clinical testing assays have been extensively reviewed elsewhere (134) and thus only briefly summarized here. The most direct assay to detect the effect of MGMT in dealkylating O^6^-MG would be in principle measuring enzyme activity (135). However, the method proposed and originally tested in glioma specimens is cumbersome for routine clinical applications and requires radioactive isotopes and availability of fresh tissue (135). After the initial studies (135,136), it has not been extensively investigated in relation to the response to alkylating agents. Immunohistochemistry of MGMT protein has been first compared with enzyme activity (137) and then correlated with response to temozolomide (2,138–140).

Initial analyses were performed by immunofluorescence in order to quantify by digital image analyses the levels of expression, whereas often the immunohistochemistry assay of MGMT protein was restricted to the count of positive cells, thus excluding the information on the real amount of expression in each cells, which is in principle the most relevant piece of information to predict the resistance to temozolomide (2,138–140). Moreover, the quantification of the percentage of positive cells could be discordant between different pathologists (141). Possibly because of these limitations, the reliability of immunohistochemistry of MGMT protein to predict response to alkylating agents in glioma has been strongly criticized (141,142) and not widely utilized in clinical practice. Although MGMT mRNA might not always represent MGMT protein expression, MGMT transcript has been tested in few studies in relation to glioma, mainly by quantitative reverse-transcription polymerase chain reaction (RT-qPCR) and less frequently by *in situ* hybridization (143–150).

Low MGMT mRNA expression has been found predictive of better response to temozolomide in at least two recent observational studies (89,150), consistently with the elevated methylation pattern of MGMT promoter. However, MGMT mRNA quantitation is not largely utilized as clinical predictive biomarker, possibly because so far these analyses on transcripts were performed in total RNA obtained from fresh tissue samples during neurosurgery (89,150), in order to cope with the instability of RNA. Therefore, more clinical observations on MGMT mRNA and clinical response to temozolomide and the feasibility of performing these analyses either from fresh tissue or from the routine tissue slices of formalin-fixed paraffin-embedded samples will be useful. Thus, for practical reasons mainly related to the stability of DNA, the most widely utilized clinical assay to estimate MGMT expression levels is the analysis of MGMT promoter methylation (151,152). Non-quantitative methylation-specific polymerase chain reaction (MS-PCR) assay after bisulfite conversion of the MGMT promoter has been utilized in the first clinical studies investigating the predictive role of MGMT methylation in response to temozolomide (6,34,38,41,49,52,60,68,149,153–157). MGMT promoter methylation has been also extended from a non-quantitative to a quantitative assay using pyrosequencing technique (158,159).

Noteworthy, the quantitative MGMT methylation, as assessed by pyrosequencing, has been correlated with progression-free survival and overall survival of patients treated with temozolomide. Patients stratified in ranges of percentage of MGMT methylation were significantly correlated with clinical outcome (50), which is consistent with an inverse correlation between promoter methylation and protein expression. Further applications of the quantitative MS-PCR assay in large clinical trials confirmed its reliability (72,74,75). Importantly, different method comparisons concluded that non-quantitative MS-PCR assays are scarcely reproducible within and between laboratories (55,160–163), whereas at the present time quantitative MS-PCR by pyrosequencing technique has become the technique of choice for clinical routine applications (164).

In summary, the most widely utilized assay to analyze the levels of expression of MGMT gene in clinical routine are based on the indirect analysis of MGMT promoter methylation. Evidence are growing on the need of stratifying patients in terms of quantitative methylation, utilizing reliable and reproducible techniques such as pyrosequencing. Further observations on quantitative assays for MGMT mRNA and quantitative MGMT methylation levels will accomplish the role of MGMT expression as biomarker predicting the response to alkylating chemotherapy, in order to provide more rational bases for decisions on the therapeutic options available for patients affected by glioma.

## 7. Manipulating MGMT expression to improve first line therapy of glioblastoma

Glioblastoma is the most common primary tumor of the central nervous system, accounting for 12–15% of all intracranial tumors and 50–60% of gliomas (165). Patients die within a few months if untreated and surgery followed by radiotherapy in addition to temozolomide prolongs median survival to 12–15 months, although disease progresses within 6–9 months, with 2-years survival <25% (165). The exponential increase of available anti-cancer targeted therapies provides new hope in improving the prognosis of these patients, since repositioning different drugs tested in other cancers to glioblastoma patients are under intensive investigation (166). For instance, anti-angiogenic targeted therapy with bevacizumab was found to increase the median progression-free survival of 4.4 months (167,168). Considering that reduced expression or silencing of MGMT gene due to promoter methylation in glioma specimens increased median overall survival after temozolomide of 6.4 months (34) and of 15.1 months comparing the group of non-methylated versus that of highly methylated tumor samples (50), reduction of MGMT expression in patients presenting non-methylated MGMT promoter could be of relevant benefit in relation to the pace of the advancements in the therapy of this specific malignant tumor.

Temozolomide itself has been shown to partially deplete the extent of expression of MGMT protein in tumor specimens of one patient affected by metastatic melanoma (2), possibly as a result of the inactivation and degradation of one molecule of MGMT protein each cycle of dealkylation of O^6^-MG, but the large series of clinical trials performed in patients affected by glioblastoma considering MGMT methylation indicated this mechanism is not sufficient in routinary clinical applications aimed to overcome the resistance to temozolomide in MGMT unmethylated glioma cells (38,47,58,60,71,74,75,86). Different alternative small organic molecules to bypass the limitations of temozolomide in MGMT unmethylated patients have been devised, as reviewed extensively elsewhere (169), such as 1,3-bis(2-chloroethyl)1-nitrosourea (BCNU), also known as carmustine, or 3-[(4-amino-2-methyl-5-pyrimidinyl) methyl]-1-(2-chloroethyl)-1-nitrosourea hydrochloride (ACNU), also known as nimustine. However, the frequent severe hematological side effects of these drugs, such as thrombocytopaenia and neutropenia in 18–23% of patients, are presently limiting the application of these drugs in patients with glioblastoma (169).

A further alternative was to sensitive tumor cells to temozolomide by concomitant use of the pseudosubstrate O^6^-benzylguanine (O^6^-BG) which also depletes MGMT by activating its ‘suicidal’ dealkylation mechanism (170). However, a Phase II clinical trial with temozolomide and concomitant O^6^-BG produced grade 4 hematological adverse events in 48% of the patients, halting further attempts to use this concomitant therapy (171). Thus, alkylating drugs alternative to temozolomide or MGMT pseudosubstrates to bypass or potentiate temozolomide are presently under scrutiny in terms of advantages for patients with unmethylated MGMT gene and suggest the need of exploring other approaches.

The present knowledge on the mechanisms of regulation of MGMT gene expression at different transcriptional and post-transcriptional levels could be utilized to design innovative tools to manipulate its expression. For instance, RNA-based molecules and analogues, locked nucleic acids (LNAs) and peptide nucleic acids (PNAs), are novel tools to upmodulate or downmodulate miRs expression (172–176), by molecular mimicry or competitive sequestration, respectively, with potential experimental therapeutic applications on post-transcriptional gene expression modulation, in principle feasible also in glioblastoma models (130).

## 8. Conclusions

MGMT expression in patients affected by glioblastoma is a double-edged sword as low levels favour cancer pathogenesis by affecting repair of DNA from environmental alkylating agents whereas high levels are responsible for the resistance to the most effective drug presently utilized, the alkylating molecule temozolomide.

**Figure 3:**
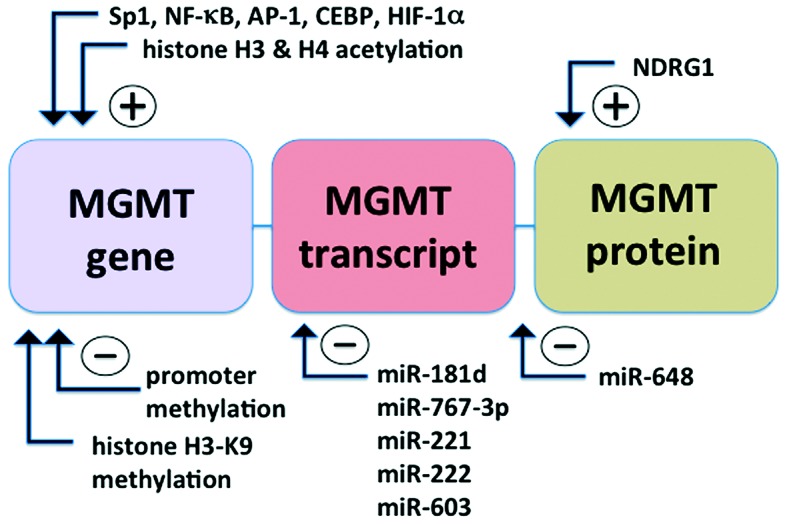
Summary of different known modulators of MGMT gene expression. MGMT expression is increased by different nuclear transcription factors (Sp1, NF-κB, AP-1, CEBP and HIF-1α) (8–13), together with the acetylation of histones H3 and H4 (18) and the stabilization by binding of N-myc downstream regulated gene 1 (NDRG1) protein (17). On the contrary, MGMT expression is downregulated by different mechanisms, namely methylation of the CpG islands in the promoter (25), di-methylation of histone H3K9 (20), degradation of mRNA by miR-181d, -767-3p, -221, -222, -603 (100,106,114,122), interference with protein translation by miR-648 (106).

Some of the molecular pathways of regulation of expression of MGMT gene have been now cleared, as summarized in Fig. 3, being the post-transcriptional regulation by miRs still an open field of investigation. MGMT expression is widely utilized in clinical practice as a predictive marker for the response to alkylating chemotherapy, and quantitation of methylation of MGMT promoter by pyrosequencing is the method of choice to identify non responders to temozolomide that can take advantage of alternative second line targeted therapies.

Artificial manipulation to silence MGMT expression in patients with unmethylated MGMT promoter to be treated with temozolomide should be explored with both innovative chemical inhibitors of MGMT function and novels molecular biology tools aimed to reduce MGMT expression by targeting MGMT mRNA stability or MGMT mRNA translation.

## Abbreviations

MGMT: O^6^-methylguanine-DNAmethyltransferase
O^6^-MG: O^6^-methylguanine
O^6^-BG: O^6^-benzylguanine
TMZ: temozolomide
miR: microRNA
MLH1: MutL homolog 1
MSH2: MutL homolog 2
MSH6: MutL homolog 6
PMS2: postmeiotic segregation increased 2
HIF-1α: hypoxia inducible factor-1α
Sp1: specificity protein 1
NF-κB: nuclear factor for polypeptide gene enhancer in B cells
c/EBP: CCAAT/enhancer binding protein
AP-1: activator protein 1
AP-2: activator protein 2
NF-IL6: nuclear factor for interleukin-6
ER-α: estrogen receptor-α
BMP2: bone morphogenetic protein 2
NDRG1: N-myc downstream regulated gene 1
HDAC: histone deacetylase
HSP90: heat shock protein 90
MeCP2: methyl-CpG binding (MCB) protein 2
TCGA: The Cancer Genome Atlas
EGFR: epidermal growth factors receptor
MS-PCR: methylation-specific polymerase chain reaction
LNA: locked-nucleic acids
PNA: peptide nucleic acids
BCNU: carmustine, 1,3-bis(2-chloroethyl)1-nitrosourea
ACNU: nimustine, 3-[(4-amino-2-methyl-5-pyrimidinyl) methyl]-1-(2-chloroethyl)-1-nitrosourea hydrochloride
